# Visuo-postural dependency index (VPDI) in human postural control

**DOI:** 10.1186/s13102-021-00235-x

**Published:** 2021-01-26

**Authors:** Alessander Danna-dos-Santos, Maria M. Ribeiro dos Santos, Alessandra T. Magalhães, Vinicius S. Cardoso, Patricia Driusso, Luis Mochizuki, Adriana M. Degani

**Affiliations:** 1 Laboratory for Advances in Rehabilitation Sciences, Department of Physical Therapy, Western Michigan University, 1903 W Michigan Av., Office # 3454, Kalamazoo, MI 49008-5383 USA; 2 Brunel University, West London, UK; 3 BioSignal Laboratory, School of Physical Therapy, Federal University of Piauí, Parnaíba, PI Brazil; 4 Physical Therapy Department, Federal University of São Carlos, São Carlos, SP Brazil; 5 School of Arts, Sciences and Humanities, University of São Paulo, São Paulo, SP Brazil

**Keywords:** Visual perception, Visual dependency, Postural control, Aging

## Abstract

**Background:**

Computerized stabilometry has been utilized to investigate the effect of vision on the neuromechanisms of human postural control. However, this approach lacks operational methods to quantify visual dependency during upright stance. This study had three goals: (1) To introduce the concept of visuo-postural dependency indices (*VPDI*) representing balance sway characteristics in multiple analytical domains (spatial, temporal, frequency, and structural), (2) To investigate the age and gender effects on *VPDI*s, and (3) To investigate the degree of relationships between *VPDI* and both subjective visual vertical and horizontal perception (*SVV* and *SVH*, respectively).

**Methods:**

102 participants (16 to 80 years old) performed bipedal stances on a force platform with eyes open and closed. Response variables included the *VPDIs* computed for each postural index. In addition, 29 participants also performed *SVV* and *SVH* assessments.

**Results:**

Fifteen *VPDI*s showed to be robust indicators of visual input modulation, and the variation across their magnitudes of modulation revealed a non-homogeneous response to changes in visual stimuli. Gender and age were not found to be a significant factor to *VPDI* modulation.

**Conclusions:**

*VPDIs* revealed to be potential measures capable to quantitatively assess visuo-postural dependency and aid the assessment of fall risks and balance impairments.

## Background

Body spatial orientation depends directly on a precise and continuous integration of visual, vestibular, and somatosensory inputs. The integration of these inputs supports the central nervous system (CNS) to create a time-to-time neural representation of the body configuration, and its relation with the surrounding environment [1, 2]. The accuracy of this internal representation is essential for the implementation of successful corrective adjustments to internal and external mechanical forces applied to the axial skeleton. The visual system, for example, uses fine oculomotor movements (such as smooth pursuit and saccadic movements) along with peripheral visual perception to provide a reference for the body’s verticality, head/body orientation, and body’s relative velocity to the visual world [3–5].

The relative importance of visual inputs on postural control is commonly referred to as visual dependency, and its applications in both laboratory and clinical settings have grown in importance. This importance is rooted to its potential role on improving predictive models of higher risks of falling in adults. Historically, the term visual dependency was coined based on Witkin and Asch (1948), who introduced the analog rod-and-frame test as a subjective visual procedure to estimate one’s degree of reliance on visual information for spatial orientation [6]. An important find was the larger levels of perceptual errors in a percentage of the healthy persons studied. These results suggest that part of our healthy population is naturally prone to higher risks of falling when their vision is impaired and/or obstructed. Although this higher risk of falling may be subclinical at younger ages, they are likely amplified by aging and/or neurological conditions. Based on this rationale, indices quantifying degrees of visual dependency could be used as an early indicator for increased fall risk. However, testing this rationale depends on the development and investigation of a multi-dimensional panel of indices representing multiple characteristics of human bipedal vertical control.

Along the past decades, the analog rod-and-frame test has been replaced by computerized methods with improved accuracy. For example, individuals are asked to adjust a projected laser bar, so the bar is perceived as either in its most vertical or horizontal position. The angular error between the bar placement and the true vertical or horizontal is computed (subjective visual vertical, *SVV*, and subjective visual horizontal, *SVH,* respectively). This valid otoneurologic clinical test has been successfully used to investigate the integrity of visual and vestibular otolithic information in different health conditions [7–9]. Despite improved accuracy and easy application, *SVV* and *SVH* tests have been limited to cases of severe impairments, such as vestibular neuritis, cerebellopontine angle tumor, posterior canal benign paroxysmal positional vertigo, and other peripheral and central vestibular lesions [10–17]. This limitation has hindered its utilization in mild and moderate cases. Therefore, less severe visual and vestibular impairments may go undetected and untreated.

Considering ample evidence suggesting human postural control as a complex series of neurophysiological processes involving several cortical and subcortical structures [18], the univariate nature of *SVV*/*SVH* testing outcomes seems to restrict its sensitivity. A potential method to improve *SVV* and *SVH* outcomes is the analysis of the body’s sway behavior (computerized stabilometry). This assessment is based on the recording of the body’s centre of pressure (COP) coordinates on a force platform during the execution of quiet bipedal vertical stance [19, 20]. Once recorded, these signals are submitted to computational procedures for the extraction of multiple indices corresponding to characteristics belonging to multiple analytical domains, i.e., spatial, temporal, frequency, and structural domains [21, 22]. In fact, this principle has been utilized to investigate the effect of vision on postural control by analyzing body sway behavior during quiet stance with eyes open and closed [23–27]. Despite their importance, these investigations were not designed to establish an actual index representing the visual dependency during postural control, nor its potential modulation across the lifespan. Such gap in the scientific literature remains.

A plausible solution to overcome this gap is the development of postural indices dedicated to the quantification of visual dependency. For example, the computation of ratios between indices obtained from different conditions of visual inputs availability could be a potential marker for visual dependency. Under this approach, individuals showing larger visual dependency would likely develop ratios departing from the normative. The use of postural index ratios was introduced by Nashner and Peters (1990) as a concept for the sensory organization testing where major sensory modalities are manipulated for the establishment of ratio-based scores [28]. This method has been clinically used in the past decades. However, the ratios and scores (i.e. maximum amplitudes of body sway and COP’s shortest distances to the base of support’s boundary) still represent only a fraction of all indices reported in laboratory as sensitive to modulation of visual inputs [28–30].

The present study was designed to bridge this gap. Here, we introduced the use of a set of *Visuo-Postural Dependency Index* (*VPDI*) representing the normalized differences calculated for multiple variables of interest recorded via computerized stabilometry, and under different conditions of visual inputs availability. We investigated the modulation of each *VPDI* to establish their ability to capture the effects of changes in visual inputs, their potential modulation across the lifespan, their correlations to *SVV* and *SVH* testing results, and a potential gender effect. Our main hypothesis is centered on the expectancy that *VPDIs* representing multiple analytical domains would be sensitive to the modulation of visual inputs. We also hypothesized that visual dependency would decrease with age. This study represents a logical progression on the study of visuo-postural dependency by providing a comprehensive panel of postural behavioral quantities and establishing an initial set of normative data across the adult life span. Moreover, the present study will offer an initial analysis of the potential relationship between *SVV/SVH* testing and stabilometric indices.

## Methods

### Participants

102 participants, aging 16 to 80 years old, participated in this study (75 females; mean height 166.4 (SD 11.4 cm), mean weight 64.7 (SD 12.6 kg). This study was performed in accordance with the Declaration of Helsinki and approved by the Institutional Review Board of the University of Montana (Missoula, MT. USA). Procedures, associated risks, and potential benefits of participation were explained to each participant and written informed consent was obtained prior to commencement of the study. Exclusion criteria for participation included history of falls within past six months, history of head trauma, traumatic brain injury, cerebral vascular accident, seizure disorders, substance abuse (drugs, alcohol, or controlled medication), peripheral neuropathy, acute upper or lower extremity injury, metallic implants in spine or extremities, neurosurgery, and abnormalities of cranial nerve functions.

### Apparatus

#### Quiet stance (VPDI) recording

A force platform (AMTI BP400600, AMTI Inc., USA) was used to acquire components of the ground reaction force (GRF) and moments of force around the frontal and sagittal axes. These signals were used to compute the body’s center of pressure (COP) coordinates in anterior-posterior and medial-lateral directions (COPap and COPml, respectively) as:

1 $$ {COP}_{ap}=\frac{-{hF}_x-{M}_y}{F_z} $$

2 $$ {COP}_{ml}=\frac{-{hF}_y-{M}_x}{F_z} $$

where *h* is the height of the base of support above the force plate, *F*_*x*_ is the anterior-posterior component of the GRF, *F*_*y*_ is the medial-lateral component of the GRF, *F*_*z*_ is the vertical component of the GRF, *M*_*x*_ is the moment of force around the sagittal axis, and *M*_*y*_ is the moment of force around the frontal axis. All signals were sampled at 100 Hz with 12-bit resolution.

#### SVV and SVH perception recording

An I-Portal system (Neurokinetics, USA) was used to assess participant’s *SVV* and *SVH* perception. Static *SVV* and *SVH* are valid otoneurologic tests to assess the perception of gravitational vertical and horizontal respectively [31]. Absolute limit *SVV* and *SVH* deviation in healthy individuals is around 2.0° to 2.5°. These values are considered reliable for both research and clinical use [32–36].

### Experimental procedure

#### Quiet stance (VPDI) recording

**Fig. 1:**
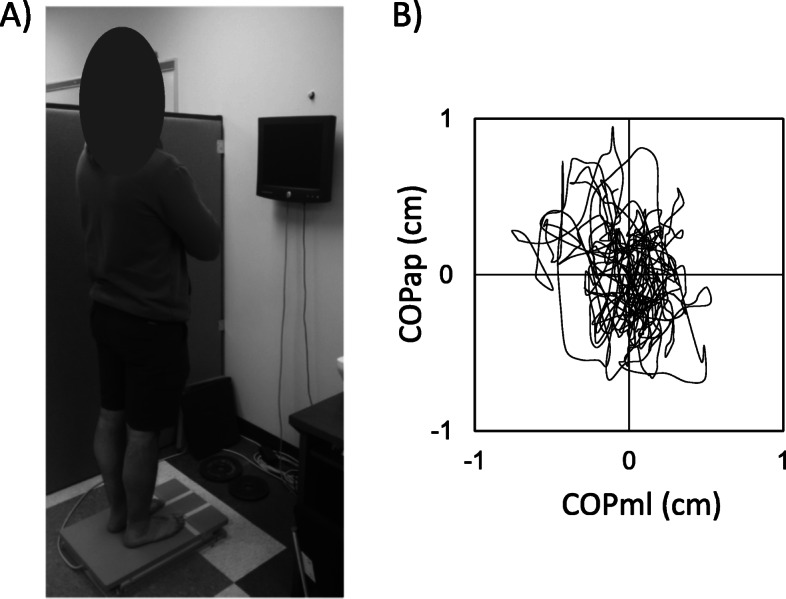
Panel **a**: Participants positioning during Bipedal Eyes Open (BEO) and Bipedal Eyes Closed (BEC) trials. Panel **b**: Representative example of posturographic recordings showing the migration of the center of pressure (COP) coordinates in anterior-posterior (COPap) and medial-lateral (COPml) directions during 120 s of data recording

For the quiet stance recordings, all participants were barefoot and asked to stand quiet for 120 s on the top of the force plate with eyes either open (BEO - bipedal eyes open, Fig. 1a) or closed (BEC - bipedal eyes closed). Feet were placed in parallel and 15 cm apart. Consistency of foot placement across participants was achieved by markings on the surface of the force plate. Participants were instructed to cross their arms against their chest while remaining as vertical as possible. During BEO condition, participants focused their vision on a physical static point placed at eyes level and 1.5 m away. During BEC condition, participants kept their eyes fully closed. To avoid any transient effects during the initial moments of a trial, data recording was initiated five seconds after the initial position was adopted by the participant. Further description of these procedures can be found in previous reports [21, 22, 27].

#### SVV and SVH perception recording

**Fig. 2:**
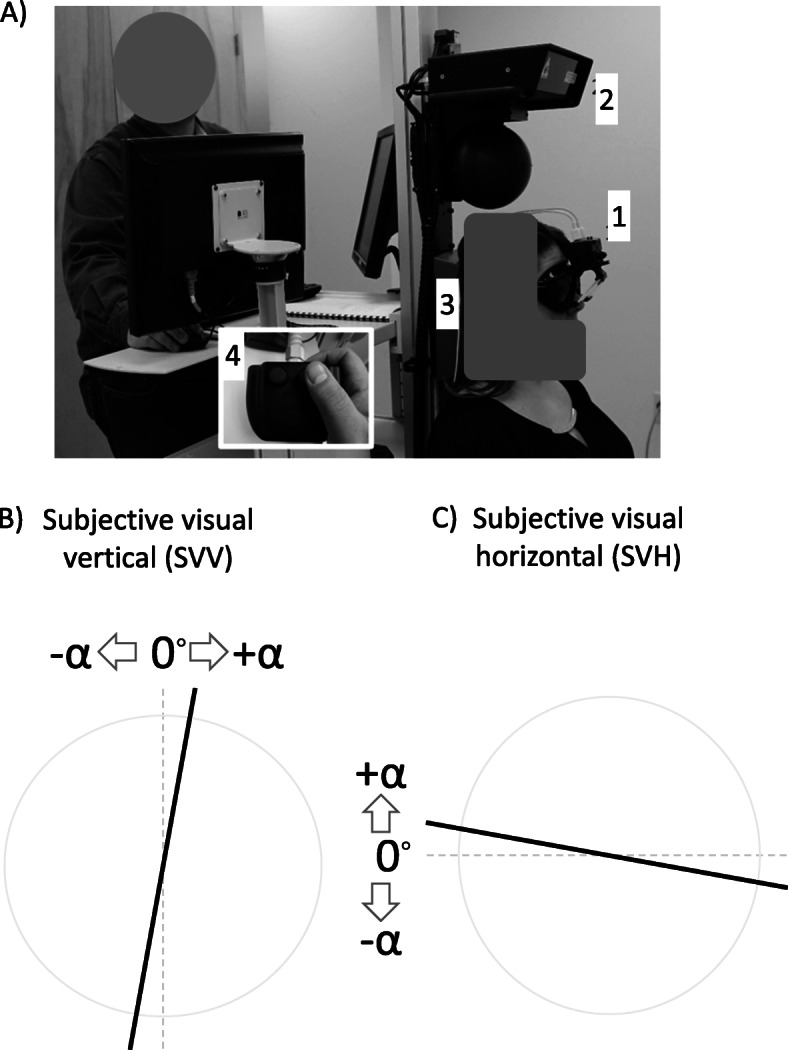
Panel **a**: Example of positioning during subjective visual vertical and horizontal (*SVV* and *SVH*, respectively) tests: (1) Eyewear utilized to restrict visual field to 110°, (2) Laser emitter, (3) Head rest, and (4) Remote control used for rotation of laser bar. Panels **b** and **c**: Schematic representation of participants’ view of the laser bar (thick trace). Dashed traces and the error (α angle) are shown for illustration purposes. They were not visible to participants. Sizes and proportions have been modified for illustration purposes

*SVV* and *SVH* perception were recorded in a representative subset of 29 female participants who were instructed to wear contoured goggles in a darkened room. They were instructed to remain seated with their head in anatomical position against a headrest (Fig. 2). A laser emitter was utilized to project a 16-cm-long monochromatic laser bar at eye level and 1.5 m ahead the participant. For both *SVV* and *SVH* tests, stimuli were designed to be offset from either the vertical or horizontal line by angular distances ranging from 5° to 35° in both clockwise and counterclockwise directions. Participants were provided with a response pad to adjust the laser bar position as vertical or horizontal as possible according to their perception. 12 trials for each *SVV* and *SVH* tests were performed in a random fashion. No time constraints for completing each trial were stipulated. The residual angle α was calculated between the true vertical or horizontal line and the final position of the laser bar (Fig. 2). The sensitivity of the system was 0.1°. Further description of these procedures can be found in a previous report [37]. Average duration of the experimental session was 20 min (SD 10 min).

### Data processing

#### Quiet stance (VPDI) data processing

COP coordinates from the force plate were analyzed off-line with customized routines (BalanceLab vs 3.0, Synergy Applied Medical and Research Inc., USA). COPap and COPml coordinate signals were down sampled to 10 Hz and detrended by the mean of each time series. This procedure allowed to position COP coordinates at the center of an *xy* coordinate system and draw any comparisons of basic COP features across participants. The following postural indices were computed: area of COP (*COP_area*), length of COP displacement (*COP_total_length*), maximum amplitude of COP displacement in each direction (*COP_range_ap* and *COP_range_ml*), root mean square estimations of COP signals (*COP_rms_ap* and *COP_rms_ml*), mean velocities of COP displacement (*COP_total_mean_vel*, *COP_mean_vel_ap*, and *COP_mean_vel_ml*), mean jerkiness of COP displacement (*COP_total_mean_jerk*, *COP_mean_jerk_ap*, and *COP_mean_jerk_ml*), median frequency of COP displacement (*COP_median_freq_ap* and *COP_median_freq_ml*), amount of regularity and predictability of COP displacement in time quantified by sample entropy of COP signals (*COP_sent_ap* and *COP_sent_ml*), and degree of asynchrony or dissimilarity between COPap and COPml signals in time quantified by cross sample entropy (*COP_crosssent*).

*COP_area* was defined similarly to procedures employing the sector formula of Leibniz previously described and used in the literature [21, 22, 37]. *COP_total_length* was computed as the total length of the COP displacement during the whole stance trial. The maximum amplitudes of the COP displacement in each direction (*COP_range_ap* and *COP_range_ml*) were computed by the difference between their maximum and minimum coordinates recorded. *COP_total_mean_vel*, *COP_mean_vel_ap*, and *COP_mean_vel_ml* were computed as the length of the COP trajectory divided by the duration of the trial. *COP_total_mean_jerk*, *COP_mean_jerk_ap*, and *COP_mean_jerk_ml* represented the time rate of COP acceleration. They were computed as the third derivative of the COP position with respect to time. *COP_median_freq_ap* and *COP_median_freq_ml* were computed as the median frequency of the COP spectral power in each direction. *COP_sent_ap* and *COP_sent_ml* were computed through estimations of the correlation, persistence, and regularity of the COP signal in time. Smaller sample entropy estimates indicate many repetitive patterns of COP fluctuation in time, whereas larger estimates indicate a more irregular, random, and unpredictable pattern. *COP_crosssent* was computed as an estimative of the correlation, persistence, and regularity in time between the COP signal in the anterior-posterior and medial-lateral directions. Higher estimates indicate larger levels of asynchrony of postural sway between the two directions, whereas lower estimates indicate more co-dependence [21, 22, 38]. Each postural index obtained during BEO and BEC were pair-wise subtracted and normalized as follows:

3 $$ VPDI\ \left(\%\right)=\Big[{\left( BEC- BEO\Big)/ BEO\right]}^{\ast }100 $$

Under this approach, a *VPDI* represents the normalized effect of vision on one variable of interest. This value can be either zero, positive, or negative. Zero values are interpreted as a null effect of vision to postural sway behavior. Positive and negative values are interpreted as either positive (beneficial) or negative (detrimental) effects of vision to postural control, respectively.

#### SVV and SVH perception data processing

Regarding static *SVV* and *SVH* tests, across-trials averages of the error in degrees (residual angle α) from each test were computed for each participant. The absolute residual angle α (*SVVα* and *SVHα*) was calculated as a dependent variable, representing the *SVV* or *SVH* perception error.

### Statistical approach

Mann-Whitney U tests were run to test the gender effect into all *VPDI*s. Due to the lack of a gender effect (Table 1), all data recorded from males and females were combined into a single sample set. Medians and quartiles for each *VPDI* are presented. One-sample Wilcoxon tests were employed to test the null hypothesis: *VPDI* equal zero (no *Vision* effect). Pearson correlation tests were applied to quantify the linear correlation level between participants’ age (*Age*) and each *VPDI*, as well as *Age* and *SVVα* or *SVHα*. A Pearson correlation test was also used to establish the linear correlation level between residual angles from subjective visual tests *SVVα* or *SVHα* and each *VPDI*. Statistical tests were performed using the IBM SPSS statistics software suite (version 22, IBM® SPSS®). A significance level of *p* <  0.05 was established and a Bonferroni’s pair-wise correction was applied to account for multiple comparisons.

**Table 1:** Median and quartiles of Visuo-Postural Dependency Index (*VPDI*) across participants (*n* = 102) for each postural index computed from center of pressure (COP) signals recorded during bipedal stance with eyes opened and closed. Results from Wilcoxon One-Sample test (visual input effect), Mann-Whitney U test (gender effect), and Pearson correlation test (age correlation) are presented

	vpdi (%)	Visual Input Effect (wilcoxon one-sample test)	Gender Effect (Mann-Whitney U test)	Age CORRELATION (Pearson test)
	Median (Q1, Q3)	*p*-value	U, z (*p*-value)	*r* (*p*-value)
*VPDI* COP_median_freq_ap (%)	45.87 (5.64, 96.52)	< 0.001*	1018.0, − 0.060 (0.952)	− 0.18 (0.065)
*VPDI* COP_mean_vel_ap (%)	40.58 (18.20, 59.24)	< 0.001*	912.0, − 0.855 (0.393)	− 0.29 (0.003)
*VPDI* COP_area (%)	40.50 (5.02, 92.42)	< 0.001*	891.0, − 1.012 (0.311)	− 0.15 (0.140)
*VPDI* COP_total_mean_vel (%)	30.41 (14.73, 50.18)	< 0.001*	881.0, − 1.087 (0.277)	− 0.28 (0.005)
*VPDI* COP_range_ap (%)	25.38 (2.66, 48.28)	< 0.001*	922.0, − 0.780 (0.435)	− 0.11 (0.292)
*VPDI* COP_mean_jerk_ap (%)	23.70 (7.33, 38.29)	< 0.001*	968.5, − 0.431 (0.666)	− 0.29 (0.003)
*VPDI* COP_total_mean_jerk (%)	17.79 (5.51, 30.39)	< 0.001*	934.5, − 0.686 (0.493)	− 0.27 (0.006)
*VPDI* COP_sent_ap (%)	16.90 (− 0.08, 39.68)	< 0.001*	1011.50, − 0.109 (0.913)	− 0.20 (0.047)
*VPDI* COP_rms_ap (%)	13.80 (− 3.05, 37.65)	< 0.001*	938.0, − 0.660 (0.509)	− 0.07 (0.497)
*VPDI* COP_mean_vel_ml (%)	13.36 (3.37, 28.74)	< 0.001*	915.0, − 0.832 (0.405)	− 0.17 (0.086)
*VPDI* COP_range_ml (%)	13.32 (−5.84, 40.90)	< 0.001*	991.0, − 0.262 (0.793)	− 0.14 (0.170)
*VPDI* COP_rms_ml (%)	11.99 (− 4.21, 31.62)	< 0.001*	891.0, − 1.012 (0.311)	− 0.15 (0.145)
*VPDI* COP_total_length (%)	11.44 (− 1.6, 32.22)	< 0.001*	907.0, − 0.889 (0.372)	− 0.10 (0.324)
*VPDI* COP_median_freq_ml (%)	10.19 (−17.82, 41.46)	0.002*	961.0, − 0.487 (0.626)	− 0.08 (0.421)
*VPDI* COP_mean_jerk_ml (%)	8.87 (0.91, 17.60)	< 0.001*	906.0, − 0.900 (0.368)	− 0.17 (0.096)
*VPDI* COP_sent_ml (%)	0.99 (−10.15, 16.30)	0.384	1012.00, − 0.105 (0.916)	− 0.01 (0.954)
*VPDI* COP_crosssent (%)	−2.96 (− 21.09, 13.02)	0.206	989.00, − 0.277 (0.781)	0.07 (0.468)

* p < 0.0029

## Results

### Quiet stance (VPDI) results

Table 1 shows the *VPDI* median and quartiles (25th and 75th) obtained across participants. These variables are ranked accordingly to their median magnitude. Most *VPDI*s revealed positive medians and the series of Wilcoxon one-sample tests showed all medians were significantly different from zero (*p ≤* 0.002), but *VPDI COP_sent_ml* (*p* = 0.38) and *VPDI COP_crosssent* (*p* = 0.20). The magnitude of significant *VPDI*s ranged from 8.87 to 45.87%, revealing an effect of visual inputs to 15 postural indices indices (VPDI cop_median_freq_ap, VPDI cop_mean_vel_ap, VPDI cop_area, VPDI cop_total_mean_vel, VPDI cop_range_ap, VPDI cop_mean_jerk_ap, VPDI cop_total_mean_jerk, VPDI cop_sent_ap, VPDI cop_rms_ap, VPDI cop_mean_vel_ml, VPDI cop_range_ml, VPDI cop_rms_ml, VPDI cop_total_length, VPDI cop_median_freq_ml, VPDI cop_mean_jerk_ml). A series of Mann-Whitney U tests did not show any significant effect of *Gender* (male vs female) on any of the *VPDIs* (Table 1). In addition, a Bonferroni’s correction was applied and no significant correlations were found between *VPDIs* and *Age* (Table 1).

### SVV and SVH perception results

Table 2 presents correlation results between either *SVVα* or *SVHα* and each *VPDI* computed for two age groups: young adults (16–30 years old) and older adults (50–74 years old). These age subgroups were selected to provide a significant distance in between the ages of the groups as well as include the early changes in postural control found in the fifth decade of life [39]. After application of Bonferroni’s correction, no significant correlations were found between *SVVα* or *SVHα* and any *VPDI.*

**Table 2:** Pearson correlation (*r*) obtained between each Visuo-Postural Dependency Indices (*VPDI*) and the absolute residual angle from either subjective visual vertical or horizontal test (*SVVα* and *SVHα*, respectively). Note: adjusted *p*-value considered for inferential statistics is 0.0029

	Pearson *r* (*p*-value)			
	Young Adults (*n* = 13)		Older Adults (*n* = 16)	
	*SVVα*	*SVHα*	*SVVα*	*SVHα*
*VPDI* COP_median_freq_ap (%)	0.12 (0.714)	− 0.07 (0.833)	0.07 (0.797)	0.08 (0.773)
*VPDI* COP_mean_vel_ap (%)	0.75 (0.005)	0.05 (0.869)	−0.18 (0.497)	−0.16 (0.563)
*VPDI* COP_area (%)	0.54 (0.069)	0.08 (0.810)	−0.30 (0.258)	−0.06 (0.818)
*VPDI* COP_total_mean_vel (%)	−0.09 (0.774)	−0.22 (0.500)	0.26 (0.329)	0.27 (0.317)
*VPDI* COP_range_ap (%)	0.76 (0.004)	0.05 (0.877)	−0.28 (0.293)	−0.22 (0.423)
*VPDI* COP_mean_jerk_ap (%)	0.02 (0.943)	−0.01 (0.965)	0.37 (0.162)	0.32 (0.224)
*VPDI* COP_total_mean_jerk (%)	−0.20 (0.526)	−0.08 (0.808)	0.25 (0.354)	0.35 (0.185)
*VPDI* COP_sent_ap (%)	−0.21 (0.522)	−0.05 (0.888)	0.03 (0.920)	0.20 (0.453)
*VPDI* COP_rms_ap (%)	−0.12 (0.721)	−0.20 (0.525)	0.65 (0.007)	0.44 (0.087)
*VPDI* COP_mean_vel_ml (%)	−0.41 (0.184)	−0.40 (0.201)	− 0.09 (0.745)	−0.19 (0.484)
*VPDI* COP_range_ml (%)	−0.42 (0.169)	−0.37 (0.241)	− 0.17 (0.523)	−0.20 (0.452)
*VPDI* COP_rms_ml (%)	−0.20 (0.527)	−0.40 (0.202)	0.13 (0.630)	−0.12 (0.661)
*VPDI* COP_total_length (%)	−0.68 (0.015)	−0.04 (0.908)	0.23 (0.395)	0.18 (0.509)
*VPDI* COP_median_freq_ml (%)	−0.17 (0.597)	−0.22 (0.488)	0.11 (0.678)	0.02 (0.947)
*VPDI* COP_mean_jerk_ml (%)	−0.76 (0.004)	−0.20 (0.542)	0.32 (0.224)	0.24 (0.366)
*VPDI* COP_sent_ml (%)	−0.19 (0.545)	−0.16 (0.619)	− 0.09 (0.742)	−0.25 (0.341)
*VPDI* COP_crosssent (%)	−0.35 (0.264)	−0.30 (0.337)	0.17 (0.532)	−0.07 (0.793)

## Discussion

### Quiet stance VPDIs and their sensitivity to visual inputs

The results uncovered here confirmed our primary hypothesis: 94.4% of *VPDIs* investigated were sensitive to the full modulation of visual inputs. This result provides evidence that multiple indices representing more than one domain of COP signal analysis should be used to assess visual dependency to postural control. Specifically, all indices investigated in the temporal, spatial, and frequency domains, and one index in the structural domain (*VPDI_COP_sent_ap*) were statistically robust to quantify the impact of visual inputs to the postural sway dynamics. This finding aligns with other investigations [40–42]. For example, Sim et al. (2018) applied a discrete wavelet transform to study the energy content of the COPap signal within frequency bands below and above 1 Hz. They have reported a temporary interruption of visual inputs can cause significant shifts in the spectral energy signal content in bands up to 1 Hz. Specific changes included reductions of energy content below 0.1 Hz and increases on frequency bands up to 1 Hz. These findings become relevant considering that the lower frequency content embedded in the COP signals has been linked to the visual neural loops involved in postural control [43–46].

The relation of specific modalities of sensory information to the energy content of the COP signal has been an area of interest in human motor control. Studies of this nature are based on the effects of neural networks complexity to the time for completion of recurring neural loops. Under this principle, more complex sensory systems (composed by longer circuits) require longer delays reaching their targeted structures. As a result, there is an addition of lower frequencies components to the COP signal. Based on previous experimentation, visual inputs are credited to add energy content to frequencies below 0.1 Hz, while vestibular and somatosensory inputs are linked to frequency bands of 0.1–0.5 Hz and 0.5–1.0 Hz, respectively [43, 44, 46]. Our results corroborate this idea. We found positive *VPDI* for the median frequency of the COP displacement in both anterior-posterior and medial-lateral directions. It was an indicative of energy increase occurring towards larger frequencies when both vestibular and somatosensory neural relays become the main sources of sensory inputs. In addition, it reinforces the robustness of the *VPDIs* on capturing subtle changes in body balance behavior due to visual input modulation.

*VPDI*s from the spatial and temporal domains (area, sway amplitude, root mean square, mean velocities, and signal jerk) were also found to be indicators of full visual modulation. Vision interruption caused participants to significantly increase their sway area, amplitude, and mean velocities. These increases were consistently more expressive in the anterior-posterior, when compared to the medial-lateral direction. These findings align with other reports showing similar effects [24, 25, 47–50]. Taken together, these studies and ours support the rationale that vision is the sensory modality producing the most reliable source of information for postural control [51–53]. Under this rationale, visual inputs are constantly fed into the CNS resulting in the construction of an internal representation of one’s relation to its surroundings (exteroception). This information is integrated to other inputs to elaborate a motor response. Finally, motor outputs are sent to postural muscles responsible for stabilization of major joints along the axial skeleton (e.g. ankle, knee, hip, and intervertebral joints). However, once vision is disrupted, the quality of exteroception information is reduced, and production of motor outputs is performed under reduced levels of certainty regarding the body’s current state and dynamics. As a result, the vertical position becomes less stable and body sway is performed with larger amplitudes and faster speeds. This observation is particularly supported by studies reporting the strong influence that motion of the visual field has on inducing phase-locked body sway motion [54–57]. The impact of low illumination on postural control has also shown significant increases in postural sway in both young and elderly participants, although this increase is significantly smaller than those observed in the eyes closed or complete darkness condition [58].

Regarding COP signal’s jerk, we observed a significant positive modulation of *VPDIs* representing COP jerk levels. This quantity is considered as an empirical measure of smoothness of posture sway and a measure of one’s ability to control motion acceleration [59]. We interpreted COP jerkiness as a sign for the presence of movement corrections executed in real time that emerges when sensory-motor integration becomes suboptimal. Partial support for this rationale originates from previous studies showing higher levels of jerk on COP signals recorded from patients suffering from Parkinson’s and Huntington’s disease [59, 60]. Despite its unclear mechanisms, such corrections are likely driven by bursts of muscular contractions aiming to decelerate the body when sway speed is elevated. Once employed, these bursts can cause short-time deviations of the COP signals from its expected pathway, and increase its degree of randomness. In fact, our results also revealed a significant positive modulation of the *VPDI* representing COPap signal’s sample entropy signaling increase on its randomness. At this time, such interpretations are only speculative and need to be investigated by dedicated experimentation.

Vision did not affect the co-dependence between COPap and COPml in healthy persons. *COP_crosssent* estimates revealed no significative changes in the synchronization between the anterior-posterior and medial-lateral *COP* displacement. Standing upright on boards with reduced support areas have affected how postural sway in both directions behave. Smaller support area (i.e. more unstable base of support) led to increased postural sway. However, a decrease in the larger dimension of the support area led to an increase in body sway above and beyond the effects of changing the smaller dimension [61]. This study suggested that anterior-posterior and medial-lateral postural sway might be more associated during unstable conditions. According to our results, it is suggested that visual-postural dependency and mechanical-postural dependency might not induce changes in the association between postural sway directions.

The degree of modulation across significant *VPDI*s was found to be remarkedly variable. *VPDI*s representing COPap characteristics revealed larger modulations compared with COPml. Based on this observation, it becomes tempting to speculate about the potential sensitivity of these measures in populations suffering from neuromuscular disorders. However, several cohorts of patients suffering from these disorders need to be studied so a conclusion could be reached. At this time, we can only expect these variations in modulation will allow the uncovering of independent patterns of visual dependency behaviors across the neuromuscular disease spectrum.

### Quiet stance VPDIs and aging

According to the literature, conventional postural indices are sensitive to age. Several investigations show older individuals progress along their late stages of life with larger, faster, more variable, and more irregular body sway in time, compared to young adults when performing bipedal stance with eyes opened [25, 47, 50, 62–64]. These changes are usually associated with the natural process of aging and its declines in sensorial, neural, and motor functioning [50, 65–68].

The use of *VPDIs* allowed us to take a step forward and assess the functional integrity of sensory-motor integration of visual inputs, while reducing the age-related effects of other sensory modalities. Under this approach, our results showed a general lack of strong relationships between age and each *VPDI*. This observation is dissonant to studies showing a decreased reliance on visual inputs to control balance after age of 65 years [69]. We believe this difference in results is related to the multivariate approach taken for our analyzes. The application of a Bonferroni’s correction to multiple comparisons reduced seventeen-fold the threshold for inferential decision. Such approach reduced our ability to capture weaker but significant effects happening to a few variables of interest. When these variables are studied independently, a negative relationship emerged between age and *VPDI* for COP velocity, jerk, and sample entropy. These correlations were found mainly on the anterior-posterior direction, suggesting that the effect of visual input on medial-lateral body sway was not dependent on the age.

It has been reported that decreases in visual reliance is due to the deterioration visual’s peripherical and central organs [70]. However, the age-related deteriorations affect all organic human systems. Therefore, the deterioration of the vestibular and proprioceptive systems are also expected, such as reduced visual acuity and accommodation, contour and depth perception, contrast sensitivity, peripheral vision, pupil size and agility, kinesthetic sensitivity, joint position sense at the ankle, and cutaneous sensation are reported in the literature [50, 71, 72]. Based on this rationale, one can speculate that aging of all sensory systems may occur at different rates and result in favoring CNS’ reliance on those systems with lower rates of deterioration or lower complexity. Currently, it is unclear the rate of progression and how this reorganization is implemented. However, this idea is supported by compounding evidence emerging from neuroimaging studies showing that the age-related effects across sensory systems differ [73, 74]. For example, deterioration of vestibular substrate has been well-documented to be linked to volume reduction of vestibular nuclei in the brainstem and reduction of cerebellar volume [73]. On the other hand, evidence of age-related changes in the visual cortex is primarily associated with functional aspects of neurons and neuronal communication in the visual system [70]. Such differences may induce loss of function at different degrees and rates and a constant necessity for the CNS to adapt its processing of sensory-motor integration during the aging years. It is possible that some may respond more efficiently to such re-organizations, while others may develop deficits resulting in higher risks or prevalence of falls. Such investigations are still to be developed.

Spatiotemporal variables are important indicators about how the CNS controls postural sway and avoid the COP reaching the limits of the body’s base of support. When such mechanisms fail, the likelihood of a fall increases. Note that when adults closed their eyes, both COP velocity and jerk increased. In addition, this increase tended to be reduced with aging (Table 2). Such associations were observed mostly for the anterior-posterior sway. These findings may be related to the mechanical properties of the axial skeleton. For example, during the execution of an upright standing posture with parallel feet position, anterior-posterior postural sway control is mostly dependent on the ability of anterior and posterior muscle groups (lower limbs and trunk) to generate the necessary torque to avoid a fall. On the other hand, medial-lateral sway is partially counteracted by the mechanical presence of two lower limbs and joints that are inherently more restricted to movements in the frontal plane. Under this rationale, one can expect that age-related muscle strength loss associated with the changes in the visual sensory inputs may have a larger impact to body-sway in the anterior-posterior direction in healthy participants.

### Quiet stance VPIDs and their relation to SVV and SVH perception

Vertical and horizontal perception were within normal values. This result was expected considering that the cohort was formed by healthy individuals with no sensory disorders. Bonferroni’s correction was applied, and no significant correlations were found between *VPDIs* and either *SVH* or *SVV,* for both young and older groups. Such results corroborated our idea of including *VPDIs* to improve current methods of visual dependency investigation. Despite the fact posturography and SVV/SVH procedures examine one’s visual dependency, they are distinct tasks representing different mechanical and cognitive constraints. For example, during the execution of *SVV* and *SVH* tasks, participants remain seated. Emphasis is shared between the mechanical maintenance of head orientation and the attention directed to a primary visual task of correcting the laser bar. On the other hand, the focus during quiet stance is on the mechanical maintenance of several body segments (trunk and lower extremities), and there is no primary visual task. Based on this rationale, one can expect these two tasks to elicit distinct neuromechanisms resulting in a few to none correlated indicators. Therefore, we interpret our findings as a step forward to understand visual dependency.

## Conclusions

The utilization of quiet stance visuo-postural dependency indices (*VPDIs*) showed to be a robust method to investigate visual input dependency to one’s postural control. Due to its multidimensional nature, this approach allows the assembly of a larger comprehensive panel of body sway characteristics. Our findings can be further examined by scientists and clinicians aiming to uncover subtle modification to the process of visual inputs integration in bipedal stance control.

## Data Availability

The datasets used and/or analyzed during the current study are available from the corresponding author on reasonable request.

## Abbreviations

CNS: Central nervous system
SVV: Subjective visual vertical
SVH: Subjective visual horizontal
COP: Center of pressure
VPDI: Visuo-postural dependency index
GRF: Ground reaction force
BEO: bipedal eyes open
BEC: bipedal eyes closed
COPap: center of pressure in anterior-posterior direction
COPml: center of pressure in medial-lateral direction
COP_area: center of pressure total area of sway
COP_total_length: center of pressure total length of sway
COP_range_ap: center of pressure signal maximum amplitude in anterior-posterior direction
COP_range_ml: center of pressure signal maximum amplitude in medial-lateral direction
COP_rms_ap: root mean square of center of pressure signal in anterior-posterior direction
COP_rms_ml: root mean square of center of pressure signal in medial-lateral direction
COP_total_mean_vel: center of pressure mean velocity
COP_mean_vel_ap: center of pressure mean velocity in anterior-posterior direction
COP_mean_vel_ml: center of pressure mean velocity in medial-lateral direction
COP_total_mean_jerk: mean jerk of center of pressure signal
COP_mean_jerk_ap: mean jerk of center of pressure anterior-posterior’s signal
COP_mean_jerk_ml: mean jerk of center of pressure medial-lateral’s signal
COP_median_freq_ap: median frequency of signal representing the center of pressure in anterior-posterior direction
COP_median_freq_ml: median frequency of signal representing the center of pressure in medial-lateral direction
COP_sent_ap: sample entropy of signal representing the center of pressure in anterior-posterior direction
COP_sent_ml: sample entropy of signal representing the center of pressure in medial-lateral direction
COP_crosssent: cross entropy of center of pressure signal
